# Higher Overall Intakes Are the Defining Feature of Dietary Intakes in NAFLD and Compared to the General Population

**DOI:** 10.3390/nu15122669

**Published:** 2023-06-08

**Authors:** Catherine Properzi, Leon A. Adams, Johnny Lo, Jill L. Sherriff, Gary P. Jeffrey, Therese A. O’Sullivan

**Affiliations:** 1 Nutrition & Health Innovation Research Institute, School of Medical and Health Science, Edith Cowan University, Joondalup, WA 6027, Australia; c.properzi@ecu.edu.au; 2 Medical School, The University of Western Australia, Nedlands, WA 6009, Australia; 3 Department of Hepatology, Sir Charles Gairdner Hospital, Nedlands, WA 6009, Australia; 4 School of Science, Edith Cowan University, Joondalup, WA 6027, Australia; j.lo@ecu.edu.au; 5 School of Public Health, Curtin Health Innovation Research Institute, Curtin University, Bentley, WA 6102, Australia; jsherriff@pointhealth.com.au

**Keywords:** non-alcoholic fatty liver disease, diet, nutritional requirements

## Abstract

We aimed to compare the dietary intakes of Australian patients with non-alcoholic fatty liver disease (NAFLD) to general Australian population intake data and determine whether the intake of any nutrient or food group was able to predict the degree of steatosis. Dietary data from fifty adult patients with NAFLD were compared to intake data from the Australian Health Survey for energy, macronutrients, fat sub-types, alcohol, iron, folate, sugar, fibre, sodium and caffeine. Linear regression models adjusting for potential confounders (age, sex, physical activity and body mass index) were used to examine predictive relationships between hepatic steatosis (quantified via magnetic resonance spectroscopy) and dietary components. The mean percentage differences between NAFLD and Australian usual intakes were significant for energy, protein, total fat, saturated fat, monounsaturated and polyunsaturated fats (all *p* < 0.001). The contribution of fat and protein to total energy intake was significantly higher in the NAFLD cohort (*p* < 0.05). No individual nutrients or food groups were strongly related to hepatic fat in the adjusted models. Higher overall consumption appears to be a major feature of dietary intake in NAFLD when compared to the general population. A whole-diet approach to NAFLD treatment and prevention is likely to be more effective than focusing on single food components.

## 1. Introduction

Non-alcoholic fatty liver disease (NAFLD) is the culmination of metabolic derangements which are characteristic of the metabolic syndrome, and it is defined by the presence of hepatic steatosis without significant alcohol consumption or other secondary causes [1,2]. The classification encompasses disease states from simple steatosis, through non-alcoholic steatohepatitis (NASH)—with or without fibrosis—to cirrhosis [2]. The prevalence of NAFLD, estimated to be as high 32.6% of the adult population worldwide and growing [3,4], represents a substantial and rising health burden. Prevalence differs significantly by geographical region, ranging from 29.8% in Europe to 56.8% in Africa [4]. Marked inter-regional differences in prevalence also exist, such as those seen between countries within the Asian region, which range from 22.2% in Japan to 34.6% in South Korea [4]. Whilst only a small percentage will develop advanced disease and progress to cirrhosis, liver failure requiring transplantation from non-alcoholic disease has grown to become the third leading cause of liver transplant in Australia and New Zealand [5]. Substantial morbidity and mortality in NAFLD from non-liver causes, such as cardiovascular disease [6], broadens the societal impacts of this disease.

A range of lifestyle factors such as smoking, BMI, physical activity, and certain dietary factors have been identified as risk factors for NAFLD [7]. Diet is also known as a key influence in the pathogenesis of NAFLD [8]; however, the specific interconnection between nutrients, foods, and patterns of intake has not been clearly elucidated. High-quality evidence for the use of any specific dietary approach in the prevention of NAFLD is limited. Attempts to group and rank dietary factors in terms of their association with NAFLD have consistently highlighted a high risk of NAFLD in those whose diets include an increased intake of fat combined with added sugars [9,10,11,12,13,14], but the results are otherwise equivocal.

Robust comparisons of quantified intake data between those with and without NAFLD are limited and uncertainty is ongoing as to which aspects of the diets of those with NAFLD place them at higher risk than the general population. A variety of sources suggest red meat [15,16,17] and soft drinks [15,16] are positively associated with NAFLD, and the consumption of a ‘fast food’ Western-type diet, consisting of energy-dense foods rich in sugar and saturated fat, has been independently associated with a higher risk of NAFLD [9]. Similarly, the consumption of low-quality ultra-processed foods is associated with markers of non-alcoholic steatohepatitis (NASH) [18], and high-quality ‘healthy diets’ are required in both prevention and management [19].

In Western countries such as Australia, where overall population intakes are largely inconsistent with healthy intake guidelines [20,21], it is unknown whether the diets of the NAFLD population differ to those of the general population to create an increased risk of metabolic ill-health. The explanation of links between intakes and the development of NAFLD is important, both in terms of prevention, and developing nutritional interventions.

In order to characterise the diets of an NAFLD cohort and determine differences from the general Australian population, we compared the usual intakes of a group of NAFLD patients to the usual intake data from the Australian Health Survey for macronutrients (protein, fat and carbohydrate), fat fractions (saturated fat, monounsaturated fat and polyunsaturated fats), iron, folate, sugar, fibre, alcohol, sodium and caffeine. We also assessed whether the intake of individual nutrients or food groups was able to predict the degree of steatosis.

## 2. Materials and Methods

### 2.1. Study Design

This comparative analysis used baseline data from 50 patients with ultrasound-diagnosed NAFLD, which was subsequently confirmed and quantified via magnetic resonance spectroscopy (MRS). Data were collected between April 2013 and June 2016 as part of a larger trial [22].

### 2.2. Subjects

Adult patients were recruited from NAFLD clinics at a Perth tertiary hospital and from private clinics, by participating gastroenterologists, as per the methods of the larger trial [22].

Subjects were included in this analysis if they met inclusion criteria for the larger randomised controlled trial [22] including the ultrasound-confirmed diagnosis of NAFLD with hepatic steatosis (quantified as >5.5% by MRS) and average alcohol consumption < 20 g/day or 140 g/week (females) or <30 g/day or 210 g/week (males).

Exclusion criteria included:

- Secondary causes of NAFLD (e.g., medication induced);
- Unstable body weight (variation > 5% within the preceding 3-month period) or current use of weight loss medications;
- Current use of pioglitazone;
- Other liver diseases or unstable diabetes (HbA1c > 8.5%);
- Decompensated cirrhosis;
- Renal failure;
- Current malignancy (aside from skin cancer);
- Inability to provide informed consent or any condition prohibiting the completion of the required assessments;
- Current smoking.

Subject numbers were based on power calculations for the larger trial [22].

### 2.3. Dietary Assessment

Usual intakes were determined for the NAFLD cohort as the mean of 7 days of dietary intake data, collected via Modified-Burke diet history interviews, carried out by a single Accredited Practising Dietitian. Quantified portion estimation photos [23] were used to estimate serving sizes for common food items.

Dietary and body weight changes within the preceding 3-month period were assessed to ensure baseline stability in these variables.

Data for the food group analyses were entered into Foodworks Professional software v9 [24] by the study dietitian. The AUSNUT 2013 and AusBrands 2017 databases were used to automatically allocate foods to food groups, based on the software’s pre-assigned categories.

### 2.4. Confounding Factors

Confounding factors were identified through their relationships to energy intake and NAFLD within the literature. Age and sex [25] and physical activity [26,27,28] from the International Physical Activity Questionnaire (IPAQ) long form [29] were included. BMI has also been associated with hepatic steatosis in a dose–response manner [30]; however, the potential for BMI to sit within the causative route between intake and NAFLD requires cautious interpretation.

### 2.5. Statistical Analysis

#### 2.5.1. Comparison to Australian Population Data

To address the question of whether intakes of the NAFLD cohort differed from those of the general Australian population, we used Australian Bureau of Statistics (ABS) data from the Australian Health Survey: Usual Nutrient Intakes [31]. These data are collected every 10–15 years and modelled to reflect what Australian adults ‘usually’ eat to allow for comparison to other data sets [20]. We used the 2011–12 intake data as this provided the most contemporaneous national data for comparison with our data, collected during 2013–16.

Data from the NAFLD cohort were coded to correspond with ABS gender and age classifications. A matched set of corresponding data were created for each nutrient. Differences between the mean intakes of ABS and NAFLD cohorts were calculated and expressed as percentage mean differences for total energy intake, nutrients, and for the percentage of total energy contributed by carbohydrate, fat, protein, and alcohol. Distributions were assessed for normality using Shapiro–Wilk tests. One-sample t-tests were performed on normally distributed data. Non-normal distributions were analysed using Wilcoxon Signed Rank tests. Exact probability values for these tests were obtained from z scores, using Microsoft Excel [32] (using norm.s.dist(z,false) function). All *p* values were analysed for false positives due to multiple analyses, using the Benjamani–Hochberg procedure with a 5% false discovery rate. 

#### 2.5.2. Relationship between Nutrients and Severity of Hepatic Steatosis

Individual nutrient data were adjusted for energy intake using the residuals method [33]. A log10 transformation was applied to baseline hepatic fat as the distribution varied significantly from normal. Potential factors to include in a multiple linear regression model were determined by examining bivariate correlations between the resulting variable and energy-adjusted nutrient variables via Spearman’s Rho method. Variables with correlation coefficients ≥0.3 were considered for inclusion.

Relationships between demographic factors and transformed hepatic fat were assessed, using Spearman’s method, to determine whether any factor was associated with degree of steatosis. Relationships between categorical variables and the adjusted hepatic fat variable were assessed via Pearson’s test or analysis of variance (ANOVA). Factors which were significantly correlated with hepatic fat were considered for inclusion as confounders. To minimize collinearity in the model, correlations between confounders were examined with the intent to remove a variable where correlation coefficients were >0.7. A multivariable linear regression was performed with transformed hepatic fat as the dependent variable.

#### 2.5.3. Relationship between Food Groups and Hepatic Steatosis

Food group variables [24] were adjusted for individual energy intake and expressed as serves per megajoule (MJ). Bivariate correlations were examined as per the correlation methodology used for nutrients.

Confounding factors were retained from the previous analysis. Food groups with correlation coefficients ≥ 0.3 with transformed hepatic fat were entered into the regression model. Multiple linear regression was performed with transformed hepatic fat as the dependent variable.

Data were analysed using IBM SPSS version 28 [34]. A value of *p* < 0.05 was used to determine statistical significance.

## 3. Results

### 3.1. Subjects

The characteristics of our NAFLD subject group and associations between demographic factors and transformed hepatic steatosis are presented in Table 1.

### 3.2. Comparison of the Nutritional Intake between NAFLD Subjects and the Australian Population

The differences between the usual mean intakes of the NAFLD cohort and the Australian intakes are presented in Table 2. Differences were significant for total energy (kJ) and all assessed nutrients and food components (*p* < 0.05), except folate equivalents, sugar and caffeine (*p* = 0.994, 0.891 and 0.270, respectively). After correction to account for multiple analyses, all tests which were significant prior to the correction remained significant, except carbohydrate (g/day) (*p* = 0.054).

The intakes of all macronutrients were higher in those with NAFLD when compared to age- and gender-matched usual Australian intakes. The percentage differences for protein and total fat were 23.9 ± 19.5% and 26.3 ± 27.3%, respectively (*p* < 0.001). Carbohydrate intakes (g/day) were also 7.5 ± 24.0% higher in those with NAFLD, although this was non-significant after adjustment. The mean energy intake was higher for the NAFLD cohort (*p* < 0.001) and was consistent with the higher intake of all macronutrients in the NAFLD cohort.

The comparison showed the consistently higher consumption of major fat sub-types (saturated, monounsaturated and polyunsaturated) in the NAFLD cohort. The percentage mean differences in intakes ranged from 18.9 ± 32.2% for saturated fat to 28.2 ± 31.9% and 37.2 ± 33.6% for mono and polyunsaturated fat, respectively.

Sugar intake was marginally but not significantly higher in the NAFLD cohort, with an absolute difference of 8.5 g per day, which equated to 0.78 + 39.3%.

Fibre, sodium, alcohol and iron also showed patterns of intake which differed significantly from those of the ABS-matched data. The fibre, sodium and iron intakes of the NAFLD cohort were significantly above those reported in the ABS cohort. Sodium was lower in the general population by 13.6 ± 21.2%, fibre 21.6 ± 22.6% and iron 19.9 ± 22.3%. The mean intake of alcohol was 0.17 ± 13.0 g per day in the NAFLD group, whilst in the matched data, intake was higher, at 15.0 ± 16.0 g per day. The difference between alcohol intakes was significant (*p* < 0.001).

The percentage of total energy intake contributed by each macronutrient within the diet was also different between the NAFLD and the ABS data. The mean difference in percentage energy derived from carbohydrate was significantly lower in the NAFLD group, whereas energy derived from fat and protein was higher in comparison to the general population (*p* < 0.001). The mean difference in the contribution of alcohol to overall energy intake was significant (*p* < 0.001).

### 3.3. Predictive Value of Nutrients in Hepatic Steatosis

Overall, individual nutrients were poorly correlated with baseline hepatic fat levels in the NAFLD group. Polyunsaturated fat intake (adjusted for energy) was the only nutrient variable which showed a relationship with hepatic fat (Rho = 0.377, *p* = 0.008).

Via multiple linear regression, polyunsaturated fat intake and hepatic steatosis (Table 3) showed that polyunsaturated fat remained significant within the regression model after adjustment for sex, age and physical activity and BMI (Table 3).

### 3.4. Predictive Value of Food Groups in Hepatic Steatosis

As with the analysis of nutrients, individual food groups were not strongly correlated with baseline hepatic fat (Supplementary Data). A weak but significant correlation between baseline hepatic fat was found for the ‘low omega-3 fish and seafood group/MJ’, Rho = 0.389 *p* = 0.006. This remained significant within the models; however, the regression models were no longer significant after the inclusion of confounding factors (Table 4).

## 4. Discussion

In this comparative analysis, intakes in our NAFLD cohort were higher than those of the general population for total energy, all macronutrients, all fat types and several other food components. The differences were significant for all nutrients except total sugar, caffeine and folate. The contributions of macronutrients towards total energy also differed significantly between the cohorts, with those in the NAFLD cohort having significantly higher energy contributions from fat and protein and lower contributions from carbohydrate.

Excess energy consumption, regardless of the source, appears to be a major driver of NAFLD [35,36]. The consumption of energy and all macronutrients at levels significantly above the matched population data clearly shows that energy consumption in our NAFLD cohort is above the age- and gender-matched usual Australian intakes. Data from other case–control studies have also shown that both obese and non-obese NAFLD subjects have caloric intakes which are significantly greater than non-NAFLD subjects [35,36].

### 4.1. Individual Foods, Nutrients and Prediction of Steatosis within Our NAFLD Cohort

We were unable to demonstrate any strong relationships between individual nutrients or food groups and the degree of steatosis. The associations between intakes of specific nutrients or food groups and the presence of steatosis, though significant in some cases, were weak. This lack of a strong association between NAFLD and any individual food or nutrient implies that overall patterns of intake, dietary quality and lifestyle factors are likely to be more important determinants of NAFLD than single nutrients within the diet. This is consistent with other evidence [19] and with the results of our larger trial [22], where two different but ‘healthy’ patterns of intake both produced significant reductions in hepatic steatosis.

The significant positive correlation between the intake of polyunsaturated fat and hepatic steatosis, seen in the NAFLD data, is surprising. Long-chain polyunsaturated fats have been associated with reduced hepatic fat in NAFLD [37,38]; however, our analysis differs in that it examined not just long-chain polyunsaturates but also total polyunsaturated fat intake. Due to shifts away from saturated fats, many ultra-processed and energy-dense ‘discretionary foods’ are prepared using polyunsaturated fats such as canola and sunflower oils. Just over 1/3 of the polyunsaturated fats consumed by Australians (1.2 of 3.2 serves) are from discretionary sources including pizzas, burgers and fish and seafood products [39]. The relationship between polyunsaturated fat intake and steatosis may be reflective of the risks of consuming a poor-quality, energy-dense diet, irrespective of the type of fat.

The weak but significant positive association seen between the consumption of low omega-3 fish and seafood and the degree of steatosis was also unexpected. The relatively high intakes of fish in the Mediterranean diet, along with evidence associating lean/white fish varieties with reduced metabolic and cardiovascular risk [40,41,42], have established a narrative that fish and seafoods confer low cardiovascular risk. While the automatic classification method used in our nutrient analysis software does not allow us to account for interactions between food or nutrient components consumed within the same dish, it is possible that ‘discretionary’ consumption such as battered and fried fish and seafood (which accounts for around 10% of discretionary unsaturated fat consumption in Australia [39]) provides a basis for the relationship between “low omega 3 fish” intake and steatosis. This again raises the point that the quality of the whole diet may be a greater factor in the pathogenesis of NAFLD than the presence of an individual food.

The examination of relationships between diet and steatosis have largely failed to find consistent links between any single nutrient and NAFLD [35] even in the case of fructose, which is widely thought to be implicated in the pathogenesis of NAFLD [43].

### 4.2. Limitations of the Current Body of Evidence

Evidence supports dietary patterns, rather than individual foods and nutrients, in the pathogenesis of NAFLD. However, evidence is largely drawn from comparison with ‘healthy’ diets, or via statistical methods used to derive food intake patterns and associated risk [9,44]. Comparisons can be problematic as we do not know whether the reference diet is the most beneficial pattern of eating in NAFLD. There is also a scarcity of evidence about the suitability of dietary quality scoring tools [45], such as Mediterranean dietary scores, used in these comparisons.

Groupings of foods based on risk-related statistical analyses are difficult to apply in practice as they do not reflect the ways in which foods fit together to form meaningful diets. Overall, researchers have yet to explain either the risk or protective benefit of such patterns, which are likely to lie in dietary quality, quantity, and metabolic load. 

### 4.3. Strengths and Limitations

The age- and sex-matched ABS data in this study provide a robust data set for the comparison of usual daily nutrient intakes [46] across a much larger cohort than matched controls and are less likely to be subject to bias introduced by any individual dietary choices. We have also used individual usual dietary intake data reflecting mean intake and employed initial checks of weight and dietary stability [47]. While this attempts to ensure that we have captured the habitual intake of each subject, it does not preclude the fact that subjects may have made changes to their diets subsequent to an NAFLD diagnosis. However, this does not detract from findings that this cohort with NAFLD consume energy and nutrients in patterns which differ from the broader community, which are concerning in their characterization of increased consumption in this cohort.

The main limitation of this study is the small sample size of our NAFLD cohort. While data for usual intakes across a larger NAFLD cohort would be of benefit in order to extrapolate to a wider NAFLD population, our analysis provides a basis for comparing dietary intakes between people with NAFLD and the general population.

## 5. Conclusions

Our findings suggest that people with NAFLD, on average, overconsume most food components examined, when compared with usual intakes of the general Australian population. Dietary improvements should be directed towards improving the quality of intake, addressing glycaemia and insulin resistance and ensuring intake is commensurate with requirements.

Our results show that individual foods and nutrients may be less important and that metabolic pathologies are driven by aspects of intake related to dietary quality and load. This is supported by previous research showing there is more than one type of ‘healthy’ diet for NAFLD [48]. Combined with differences in human genetics, epigenetics and metabolism, it is not possible to conclude that any individual nutrient or food is always protective or detrimental.

## Figures and Tables

**Table 1:** Association between hepatic fat ^c^ determined via magnetic resonance spectroscopy and demographic, health and lifestyle factors in patients with NAFLD.

N = 50	Mean	SD	Correlation (Spearman’s Rho)	*p*
Sex				
Females n (%)	24 (48.0%)		0.113 ^a^	0.436 ^a^
Age (years)	52.5	11.5	0.157	0.275
Ethnicity n (%)			0.314 ^b^	0.815
Caucasian	41 (82.0%)			
Asian	7 (14.0%)			
Other	2 (4.0%)			
Diabetes n (%)	15 (30%)		0.166	0.250 ^a^
Anthropometry				
Weight (kg)	85.0	13.4	−0.009	0.952
Waist (cm)	101.6	11.7	0.179	0.218
BMI (kg/m^2^)	30.9	4.9	0.109	0.453
Liver				
Hepascore	0.33	0.32	0.183	0.207
Liver Stiffness (kPa)	9.6	11.2	0.324	0.028
Bilirubin (µmol/L)	12.5	4.5	−0.195	0.174
ALT (U/L)	72.1	58.6	0.424	0.002
ALP (U/L)	87.2	37.6	0.053	0.720
GGT (U/L)	108.9	119.8	0.156	0.283
AST (U/L)	49.9	41.6	0.369	0.009
Cardiovascular				
Systolic BP (mm Hg)	127.5	15.4	0.008	0.955
Diastolic BP (mm Hg)	79.9	7.9	0.007	0.961
Total Cholesterol (mmol/L)	5.0	1.1	0.004	0.977
Triglycerides (mmol/L)	1.7	0.8	0.123	0.403
HDL cholesterol (mmol/L)	1.2	0.3	−0.173	0.239
LDL cholesterol (mmol/L)	3.0	1.0	−0.009	0.952
FRS	4.3	5.0	0.144	0.345
Lifestyle				
Glucose (mmol/L)	5.6	1.1	0.411	0.003
Insulin (mU/L)	14.8	9.5	0.486	<0.001
HbA1c (%)	6.0	1.0	0.261	0.067
HOMA2-IR	1.8	0.8	0.472	0.001
Activity (MET-h/wk)	55.8	59.6	−0.052	0.721
QoL score (/100)	79.4	10.3	0.021	0.889

Correlations determined non-parametrically using Spearman’s Rho. ^a^ = correlation determined using Pearson’s test due to dichotomous categorical variable. ^b^ = one-way ANOVA F(3, 46). ^c^ = log 10 transformed.

**Table 2:** Comparison of Australian-population-matched data to nutrient intakes of NAFLD subjects with percentage differences.

Variable	NAFLD Cohort		Matched ABS Data		Percentage Difference (%)		Corrected *p* *
	Mean	SD	Mean	SD	Mean	SD	
Energy (kJ/day)	10,218	2183	8646	1312	15.4	17.9	<0.001
Carbohydrate (g/day)	242.9	64.8	221.3	35.7	7.15	24.0	0.054
Sugar (g/day)	108.6	45.4	100.1	13.5	0.78	39.3	0.943
Protein (g/day)	114.7	21.7	89.7	15.2	23.9	19.5	<0.001
Total Fat (g/day)	100.2	30.9	73.7	10.4	26.3	27.3	<0.001
Saturated Fat (g/day)	35.0	12.0	27.5	4.1	18.9	32.2	<0.001
Monounsaturated Fat (g/day)	40.0	14.2	28.3	4.1	28.2	31.9	<0.001
Polyunsaturated Fat (g/day)	17.6	6.5	11.3	1.3	37.2	33.6	<0.001
Fibre (g/day)	29.6	7.0	23.3	1.9	21.6	22.6	<0.001
Caffeine (mg/day) ^α^	202.9	178.0	180.0	4.25	10.3	95.9	0.304
Sodium (mg/day)	2793.4	662.8	2398.2	391.4	13.6	21.2	<0.001
Daily Folate eq. ug/day	655.2	264.0	612.4	72.1	0.04	39.3	0.994
Alcohol g/day ^α^	0.17	13.1	15.0	16.0	199.7	168.5	0.002
Iron mg/day	13.8	2.9	11.2	1.5	19.9	22.3	<0.001
% Energy from CHO	38.9	6.6	42.4	0.6	0.10	0.17	<0.001
% Energy from Fat	35.8	5.6	30.8	0.4	0.14	0.16	<0.001
% Energy from Protein ^α^	19.4	4.1	18.0	1.0	0.05	0.20	0.025
% Energy from Alcohol ^α^	0.005	3.94	5.0	0.5	1.99	1.63	<0.001

* Significance after Benjamani–Hochberg correction. ^α^ = Non-normal distribution assessed via Shapiro–Wilk test. Median (IQR) data are presented. Significance assessed non-parametrically via Wilcoxon-signed rank test using a median of 0. NB. % energy provided by other dietary components not assigned to protein, fat, saturated fat, carbohydrate and alcohol includes fibre, organic acids and polydextrose [24].

**Table 3:** Linear regression for relationship between nutrient intake and severity of hepatic fat (log transformed), controlling for gender, age, physical activity and BMI.

Model	Factors	Factor Significance	Beta (Unstandardised)	Beta (Standardised)	95% CI	Adjusted R^2^	F	*p*
1	(Constant)	<0.001	−0.807		−1.174–−0.439	−0.032	0.519	0.671
	Age	0.347	0.003	0.139	−0.004–0.01			
	Sex	0.572	0.046	0.088	−0.116–0.208			
	Physical Activity (MET-h/wk)	0.790	0.000	−0.04	−0.002–0.001			
2	(Constant)	<0.001	−0.753		−1.112–−0.395	0.42	1.519	0.214
	Age	0.522	0.002	0.097	−0.005–0.009			
	Sex	0.610	0.040	0.077	−0.116–0.196			
	Physical Activity (MET-h/wk)	0.738	0.000	−0.049	−0.002–0.001			
	Polyunsaturated fat (g/kJ/day)	0.042	0.016	0.303	0.001–0.032			
3	(Constant)	0.016	−0.771		−1.388–−0.154	0.02	1.188	0.331
	Age	0.526	0.002	0.097	−0.005–0.009			
	Sex	0.636	0.038	0.074	−0.124–0.201			
	Physical Activity (MET-h/wk)	0.743	0.000	−0.049	−0.002–0.001			
	BMI	0.944	0.001	0.11	−0.016–0.017			
	Polyunsaturated fat (g/kJ/day)	0.050	0.016	0.301	0.000–0.032			

Polyunsaturated fat and hepatic steatosis (log transformed) displayed correlation of ≥0.3, and the relationship was examined in a multivariable linear regression with age, sex and physical activity. BMI was added separately to the model due to its relationship to both steatosis and dietary intake. Analysis of residuals for all models showed that they were not significantly different from normal, as determined via Shapiro–Wilk test of normality *p* > 0.05.

**Table 4:** Linear regression for relationship between food group and severity of hepatic fat (log transformed), controlling for gender, age, physical activity and BMI.

Model	Factors	Factor Significance	Beta (Unstandardised)	Beta (Standardised)	95% CI	Adjusted R^2^	F	*p*
1	(Constant)	<0.001	−0.807		−1.174–−0.439	−0.032	0.519	0.671
	Age	0.374	0.003	0.139	−0.004–0.010			
	Sex	0.572	0.046	0.088	−0.116–0.208			
	Physical Activity (MET-h/wk)	0.790	0.000	−0.040	−0.002–0.001			
2	(Constant)	<0.001	−0.935		−1.301–−0.569	0.069	1.871	0.133
	Age	0.278	0.004	0.161	−0.003–0.010			
	Sex	0.349	0.073	0.141	−0.083–0.228			
	Physical Activity (MET-h/wk)	0.991	−7.373 × 10^−6^	−0.002	−0.001–0.001			
	Low omega 3 seafood (serves/MJ/day)	0.021	4.854	0.345	0.775–8.934			
3	(Constant)	0.001	−1.042		−1.641–−0.443	0.052	1.512	0.207
	Age	0.282	0.004	0.162	−0.003–0.011			
	Sex	0.426	0.064	0.124	−0.097–0.226			
	Physical Activity (MET-h/wk)	0.999	8.105 × 10^−7^	0.000	−0.001–0.001			
	BMI	0.649	0.004	0.067	−0.012–0.019			
	Low omega 3 seafood (serves/MJ/day)	0.022	4.851	0.345	0.730–8.971			

Low omega 3 seafood [24] and hepatic steatosis (log transformed) displayed correlation of ≥0.3, and the relationship was examined in a multivariable linear regression with age, sex and physical activity. BMI was added separately to the model due to its relationship to both steatosis and dietary intake. Analysis of residuals for all models using Shapiro–Wilk test of normality showed they do not differ significantly from normal *p* < 0.005.

## Data Availability

Publicly available datasets were analysed in this study. These data can be found here: https://www.abs.gov.au/statistics/health/health-conditions-and-risks/australian-health-survey-nutrition-first-results-foods-and-nutrients/latest-release#data-downloads. Downloaded 22 March 2018. Data presented in this study are available in within the Supplementary Materials.

## Supplementary Materials

The following supporting information can be downloaded at: https://www.mdpi.com/article/10.3390/nu15122669/s1.
